# Astrocytes in neuroinflammation and brain cancer

**DOI:** 10.1186/s43556-026-00439-y

**Published:** 2026-03-30

**Authors:** Wei Sun, Pin Chen, Xiao-Yin Xu, Jia-Qi Zhang, Wei-Lin Jin

**Affiliations:** 1 Department of Neurosurgery, Zhongshan Hospital, Fudan University, 180 Fenglin Road, Shanghai, 200032 China; 2 Department of Pharmacy, Shanghai Municipal Hospital of Traditional Chinese Medicine, Shanghai University of Traditional Chinese Medicine, Shanghai, 200071 China; 3 Central Laboratory, Nanxiang Branch of Ruijin Hospital, Shanghai Jiao Tong University School of Medicine, Shanghai, 201802 China; 4 Institute of Cancer Neuroscience, Medical Frontier Innovation Research Center, The First Hospital of Lanzhou University, The First Clinical Medical College of, Lanzhou University, Lanzhou, 730000 China

**Keywords:** Astrocytes, Neuroinflammation, Glioblastoma, Tumor microenvironment, Immunotherapy, Metabolic reprogramming

## Abstract

Astrocytes are increasingly recognized as active regulators of glioma progression rather than passive bystanders. In addition to blood–brain barrier support, metabolic homeostasis, and synaptic regulation, astrocytes undergo state transitions in response to tumor-derived cues, immune inflammation, and therapy-induced stress. We synthesize evidence from single-cell and single-nucleus transcriptomics, spatial transcriptomics, proteomics, and multiplex imaging to delineate major tumor-associated astrocyte programs across perivascular, invasive-margin, and hypoxic niches. Mechanistically, we highlight how convergent signaling networks, including interleukin-6 (IL-6) family signal transducer and activator of transcription 3 (STAT3), nuclear factor-kappa B (NF-κB), interferon, and transforming growth factor-beta (TGF-β) pathways, couple to metabolic rewiring and chromatin reinforcement to stabilize pro-tumor phenotypes and define molecular inflection points during disease evolution. We propose a 4D spatiotemporal mapping framework that integrates staged sampling with spatially resolved readouts to reconstruct astrocyte trajectories and predict therapy-induced state shifts. To accelerate translation, we separate tumor-derived analytes from astrocyte-derived response analytes within a glial liquid biopsy concept, emphasizing extracellular vesicles, cell-free nucleic acids, and state-linked protein signatures. Finally, we discuss state-aware interventions, spanning pharmacologic modulation and gene therapy, with an emphasis on implementable RNA therapeutics such as small interfering RNA (siRNA)lipid nanoparticles and central nervous system (CNS)-appropriate delivery routes, to restore protective barrier functions while limiting immune exclusion and invasion. We outline endpoint panels for in vivo validation and patient stratification, and identify priorities for clinical translation, including longitudinal sampling, spatial atlases, and combinations of astrocyte normalization with immunotherapy and radiotherapy.

## Introduction

Astrocytes represent the most abundant glial cells in the mammalian central nervous system (CNS), historically viewed as passive structural supports, often likened to the "glue" of neural circuits. This perspective originated from early histological studies but has evolved significantly with advancements in neuroscience, revealing astrocytes as dynamic regulators integral to CNS homeostasis. They form key parts of the neurovascular unit (NVU) and tripartite synapse, managing processes such as blood–brain barrier (BBB) maintenance through endfeet ensheathment and secretion of factors like sonic hedgehog (SHH), retinoic acid, and angiopoietin-1, which upregulate tight junction proteins [1–4]. Astrocytes also regulate cerebral blood flow via vasoactive metabolites [5], facilitate neurotransmitter homeostasis by clearing glutamate via excitatory amino acid transporters 1 and 2 (EAAT1/2) [6–11], provide metabolic support through the lactate shuttle and glycogen storage [12–14], buffer ions like potassium via Kir4.1 channels [15–17], and exhibit intrinsic diversity with protoplasmic and fibrous subtypes showing regional and molecular heterogeneity [18–21]. This foundational understanding of their physiological roles is crucial for interpreting their plasticity in pathology, where baseline diversity influences responses to inflammation and cancer.

The impetus for this review arises from the increasing evidence that astrocytes serve as central mediators linking neuroinflammation and brain malignancies, particularly glioblastoma (GBM), yet current literature often addresses these aspects separately, missing opportunities for integrated insights. Despite progress in glial biology, gaps persist in how inflammatory pathways are hijacked in tumors, leading to therapeutic resistance. This review bridges these gaps by synthesizing recent findings on astrocyte reprogramming, drawing from technologies like single-cell RNA sequencing to explore their dual roles—from protective guardians in acute inflammation to tumor-promoting accomplices in chronic malignancy [22–25]. By emphasizing this convergence, we aim to highlight astrocytes' contributions to tumor microenvironment (TME) dynamics and propose stromal normalization as a strategy to enhance clinical outcomes.

Key highlights include the co-option of pathways like nuclear factor-kappa B (NF-κB) and Janus kinase/signal transducer and activator of transcription 3 (JAK/STAT3) for astrocyte transformation into tumor-associated phenotypes, enabling metabolic feeding via the "reverse Warburg" effect, mitochondrial transfer through tunneling nanotubes, and immune evasion via programmed death-ligand 1 (PD-L1) and TNF-related apoptosis-inducing ligand (TRAIL) upregulation [26–36]. We also address systemic influences such as the gut-brain axis and hormonal signaling, alongside therapy-induced plasticity that exacerbates resistance [37–43]. These elements underscore astrocytes' phenotypic spectrum, from A1/A2 states in inflammation to spatially heterogeneous niches in cancer, offering targets for interventions like connexin-43 blockade and CAR-T therapies.

Against this background, this review is organized around a central premise: astrocytes are not merely reactive bystanders in diseased brain tissue, but context-dependent regulators that connect inflammatory signaling, metabolic adaptation, stromal remodeling, and therapeutic response across the continuum from neuroinflammation to brain cancer. Accordingly, we first summarize the physiological functions and intrinsic heterogeneity of astrocytes as the conceptual basis for understanding their pathological plasticity. We then examine how inflammatory activation reshapes astrocyte states and signaling outputs, with particular attention to the transition from homeostatic support to maladaptive immune and trophic programs. Building on this framework, we next discuss how these altered astrocyte states are co-opted within brain tumors to support invasion, immune evasion, metabolic coupling, and resistance to treatment. We further integrate emerging evidence on bidirectional crosstalk between astrocytes and other cellular and environmental components of the tumor microenvironment, including epigenetic regulation, hypoxia, intercellular communication, and therapy-induced reprogramming. Finally, we evaluate current and prospective therapeutic strategies that aim not simply to eliminate astrocytes, but to normalize or redirect their disease-associated states, and we conclude by outlining key priorities for future research, including spatiotemporal mapping, biomarker development, and clinically actionable state-targeted interventions.

## Role of astrocytes in neuroinflammation

Neuroinflammation is a complex, orchestrated response of the central nervous system to injury, infection, or disease. While microglia have long been considered the primary immune sentinels of the brain, astrocytes are now recognized as equally critical effectors in the neuroinflammatory cascade. Their strategic positioning at the BBB and their extensive synaptic coverage enable them to act as both sensors of danger signals and amplifiers of immune responses. This section examines the mechanisms of astrocyte activation, their signaling repertoire, and their complex, often contradictory roles in pathological environments.

### Astrocyte activation and phenotypic plasticity

In response to CNS insults—whether traumatic, ischemic, or degenerative—astrocytes undergo profound morphological, molecular, and functional changes collectively termed reactive astrogliosis [44]. Historically viewed as a graded response ranging from mild hypertrophy to scar-forming proliferation, recent advances in single-cell transcriptomics have revealed that reactive astrocytes exhibit remarkable phenotypic plasticity, adopting distinct states dictated by the specific inflammatory milieu.

A pivotal conceptual framework, introduced by Liddelow et al., categorizes reactive astrocytes into two polarized states analogous to the macrophage M1/M2 classification: A1 (neurotoxic) and A2 (neuroprotective) [22]. The A1 phenotype is induced primarily by a cocktail of microglia-derived cytokines (IL-1α, TNF-α, and C1q). In this state, astrocytes lose their ability to support neuronal survival and synaptogenesis, instead acquiring toxic functions that actively kill neurons and mature oligodendrocytes, a phenotype prevalent in neurodegenerative diseases and aging [22, 45]. Conversely, ischemia or specific repair signals can induce an A2 phenotype, characterized by the upregulation of neurotrophic factors (e.g., BDNF, VEGF) and thrombospondins, which promote neuronal survival and tissue repair [45]. However, this binary model is increasingly viewed as an oversimplification, as reactive astrocytes likely exist along a multidimensional spectrum. In chronic inflammation, for example, astrocytes may co-express markers of both A1 and A2 states or adopt unique "pan-reactive" profiles that do not fit neatly into either category [23]. Recent single-cell RNA sequencing (scRNA-seq) studies have further refined this landscape. In GBM, distinct astrocyte subtypes have been identified based on unique gene expression profiles: protoplasmic-like (AST1), oligodendrocyte- and neuronal gene-expressing (AST2), and inflammatory (AST3) reactive astrocyte subtypes. These subtypes likely reflect different stages of differentiation or responses to environmental stress [24, 25]. This plasticity underscores that astrocyte function is not fixed but is dynamically shaped by the duration and intensity of the inflammatory trigger.

### Signaling pathways involved in astrocyte-mediated inflammation

The transition from a resting to a reactive state is governed by specific intracellular signaling cascades. Two pathways stand out as master regulators of astrocyte-mediated inflammation: the NF-κB pathway and the JAK/STAT3 pathway.

#### The NF-κB pathway

NF-κB is a central driver of the pro-inflammatory program in astrocytes. Under physiological conditions, NF-κB is sequestered in the cytoplasm by inhibitory IκB proteins. Stimulation by Toll-like receptors (TLRs) or cytokine receptors, such as the interleukin-1 receptor (IL-1R) and tumor necrosis factor receptor (TNFR) triggers IκB degradation, allowing NF-κB to translocate to the nucleus [26]. TLRs, particularly TLR2 and TLR4, are key pattern recognition receptors that regulate the neuroinflammatory response in astrocytes. These receptors can be activated by microbial-associated molecular patterns (such as lipopolysaccharides), leading to downstream activation of the myeloid differentiation primary response 88 (MyD88), NF-κB, and mitogen-activated protein kinase (MAPK) pathways [27]. NF-κB activation in astrocytes induces the robust transcription of chemokines (e.g., CCL2, CXCL10), adhesion molecules (e.g., VCAM-1, ICAM-1), and pro-inflammatory cytokines. This pathway essentially converts astrocytes into immune effectors, recruiting peripheral leukocytes into the CNS parenchyma and amplifying local inflammation. In many pathologies, including glioblastoma, constitutive NF-κB activation in astrocytes is a key mechanism sustaining a chronic inflammatory microenvironment [28, 29].

#### The JAK/STAT3 pathway

While NF-κB drives inflammation, the Janus Kinase/Signal Transducer and Activator of Transcription 3 (JAK/STAT3) pathway is the primary orchestrator of reactive astrogliosis and glial scar formation. STAT3 phosphorylation is a ubiquitous signature of reactive astrocytes across virtually all CNS pathologies [30]. Functionally, STAT3 signaling promotes astrocyte hypertrophy and upregulates intermediate filaments like glial fibrillary acidic protein (GFAP) and vimentin. Crucially, STAT3-dependent scar formation establishes a physical barrier that restricts the spread of inflammatory cells and infectious agents, thereby protecting adjacent healthy tissue [31, 32]. Deletion of STAT3 in astrocytes results in widespread immune cell infiltration and exacerbated neuronal loss, highlighting its essential role in containing inflammation. However, in the context of cancer, this same pathway can be hijacked to promote immunosuppression, as detailed later [33, 34].

#### Other contributing pathways

Beyond these central pathways, the calcineurin-nuclear factor of activated T cells (NFAT) pathway helps fine-tune the inflammatory profile, particularly in response to calcium dysregulation [46, 47]. Additionally, the MAPK/ERK pathway modulates the proliferative capacity of reactive astrocytes [48, 49].

### Cytokine and chemokine production by astrocytes

Once activated, astrocytes become prolific secretory cells, releasing a diverse “secretome” that profoundly influences neighboring cells. They are a major source of chemokines such as CCL2 (MCP-1), CXCL10 (IP-10), and CXCL12 (SDF-1) in the inflamed brain. CCL2 is particularly potent in recruiting CCR2 + monocytes and microglia to sites of injury [50], a chemotactic gradient that is also critical for the accumulation of tumor-associated macrophages (TAMs) in brain tumors. Concurrently, astrocytes produce pro-inflammatory cytokines including IL-6, TNF-α, and IL-1β. IL-6, produced downstream of NF-κB and STAT3, exerts pleiotropic effects—promoting T-cell differentiation while also supporting neuronal survival depending on the signaling context [51–53]. To resolve inflammation, astrocytes also release anti-inflammatory factors like TGF-β and IL-10. While TGF-β acts as a key suppressor of microglial activation to limit CNS autoimmunity, in gliomas, astrocyte-derived TGF-β becomes a potent driver of immune evasion by inhibiting NK cells and cytotoxic T lymphocytes [54–56].

### Interactions between astrocytes and microglia

Neuroinflammation is rarely driven by a single cell type; rather, it emerges from intricate bidirectional crosstalk between astrocytes and microglia. In microglia-to-astrocyte signaling, microglia often serve as the first responders to injury. As demonstrated by Liddelow et al., activated microglia release IL-1α, TNF-α, and C1q, which act synergistically to induce the neurotoxic A1 astrocyte phenotype; in the absence of this trigger, astrocytes frequently fail to acquire fulminant neurotoxic properties [57]. Conversely, astrocyte-to-microglia signaling allows astrocytes to modulate microglial states. They secrete factors like orosomucoid-2 (ORM2), which inhibit microglial activation and migration, thereby preventing excessive neuroinflammation [58, 59]. Alternatively, astrocytic release of ATP or glutamate can activate microglial purinergic receptors (e.g., P2X7), amplifying the immune response. Advanced functional screening platforms, such as droplet-based forward genetic screening, have recently identified inhibitory signaling molecules (e.g., heparin-binding epidermal growth factor-like growth factor) transmitted from microglia to astrocytes, offering new insights into modulating this crosstalk [60]. Furthermore, intervention targeting extracellular matrix-receptor interactions using β1-integrin antagonist peptides containing arginine-glycine-aspartate(RGD) sequences has been shown to attenuate glial scar densification and promote re-expression of the astrocytic glutamate transporter GLT-1 [61], further illustrating the complexity of these interactions. In the tumor microenvironment, this crosstalk establishes a vicious cycle wherein tumor cells exploit glial interactions to foster an immunosuppressive niche [60, 62].

### Dual roles of astrocytes in neuroinflammation

The role of astrocytes in inflammation is fundamentally dualistic—a "double-edged sword" that persists in the context of brain cancer. In the acute phase of injury, astrocyte reactivity is largely beneficial and crucial for tissue preservation. They contribute to BBB repair to prevent edema, uptake excitotoxic glutamate, and form a glial scar that sequesters toxic debris and contains inflammatory cells [63, 64]. Indeed, complete ablation of reactive astrocytes exacerbates tissue damage in early stages. However, in chronic conditions, persistent reactive astrogliosis becomes maladaptive and destructive. The continuous release of pro-inflammatory cytokines sustains a toxic loop driving neurodegeneration, while A1 astrocytes contribute to synaptic stripping and oligodendrocyte death. Furthermore, the glial scar, initially protective, eventually acts as a physical and chemical barrier rich in chondroitin sulfate proteoglycans that impedes axonal regeneration and hampers drug delivery [65, 66].

**Fig. 1:**
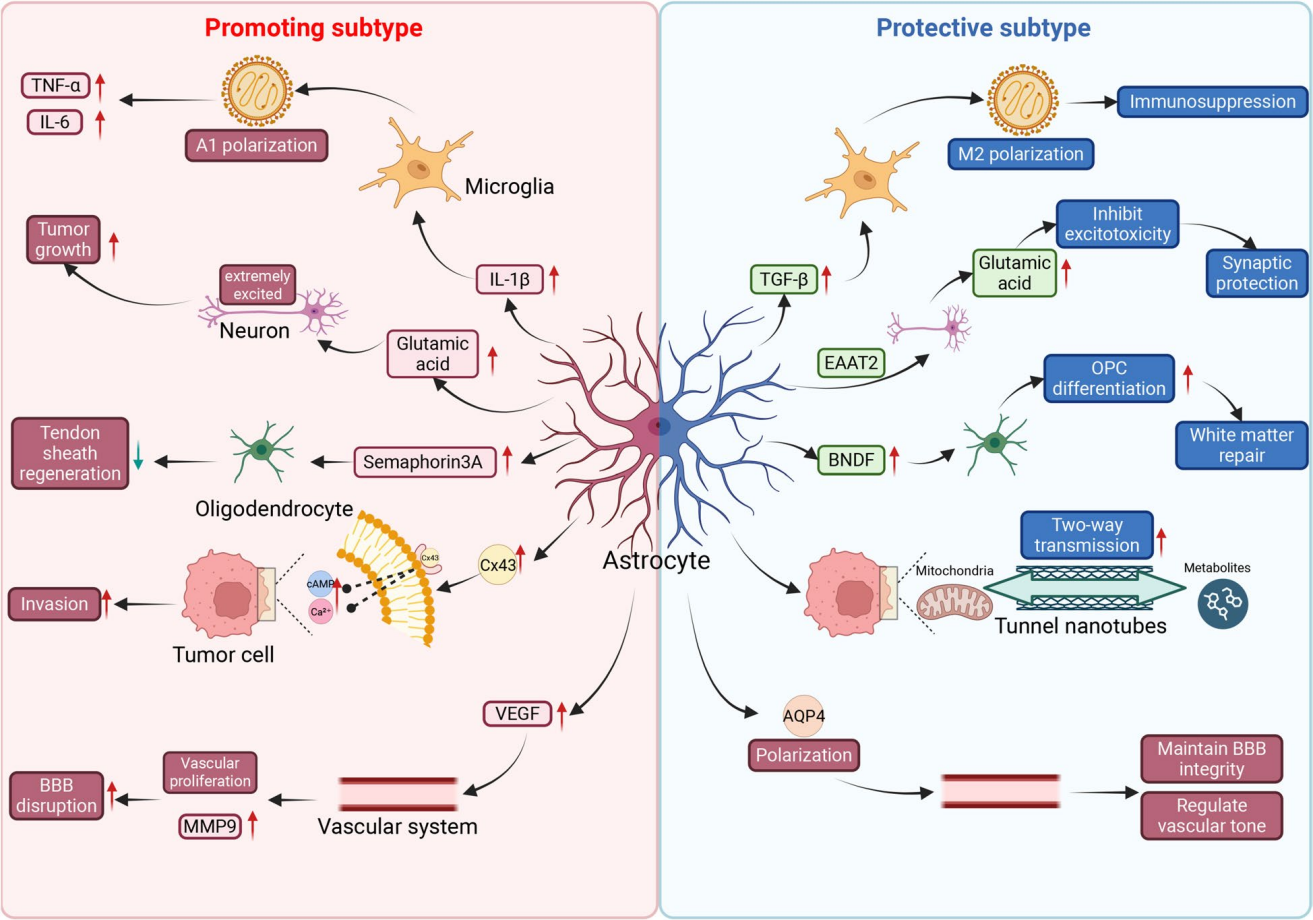
Key astrocyte interactions and functional consequences of heterogeneity within the tumor microenvironment. This figure contrasts the dual roles of heterogeneous astrocyte subtypes. The protumorigenic population (left panel) drives tumor progression by inducing pro-inflammatory A1 polarization in microglia and facilitating tumor cell invasion via connexin-43 gap junctions. Conversely, the protective population (right panel) restrains tumor progression by promoting anti-inflammatory M2 microglial polarization, protecting neurons from excitotoxicity through glutamate uptake, and maintaining blood–brain barrier integrity via polarized aquaporin-4 (AQP4) expression. This figure was created with BioRender.com. Abbreviations: AQP4, aquaporin-4; EAAT2, excitatory amino acid transporter 2; BDNF, brain-derived neurotrophic factor; Cx43, connexin-43; VEGF, vascular endothelial growth factor; MMP9, matrix metalloproteinase-9; BBB, blood–brain barrier; TNF-α, tumor necrosis factor-alpha; IL-1β, interleukin-1 beta; IL-6, interleukin-6; TGF-β, transforming growth factor-beta; OPC, oligodendrocyte precursor cell

Understanding this duality—protective in acute phases versus detrimental in chronic conditions—is essential for therapeutic development. In brain tumors, the challenge is magnified: clinicians must inhibit the tumor-promoting aspects of astrocyte activation (e.g., immunosuppression, invasion support) without compromising their essential homeostatic and barrier-preserving functions (Fig. 1).

## Astrocytes and brain cancer

While astrocytes serve as guardians of homeostasis in the healthy brain, they can be coerced into becoming accomplices in cancer progression. The tumor microenvironment (TME) of brain malignancies constitutes a complex, integrated ecosystem where reactive astrocytes—often termed tumor-associated astrocytes (TAAs)—play a dominant regulatory role. This section explores the molecular classification of astrocytic tumors and examines the ecological dynamics of the TME, focusing on the multifaceted contributions of TAAs [67].

### Classification and characteristics of brain cancers

The classification of primary brain tumors has undergone a paradigm shift with the advent of the 2021 WHO Classification of Tumors of the Central Nervous System. This updated system prioritizes molecular parameters over traditional histology, fundamentally reshaping the nosology of “gliomas” and enforcing a clear distinction between astrocytic and oligodendroglial lineages [68–70].

#### Glioblastoma, IDH-wildtype

Glioblastoma (GBM) is the most common and lethal primary malignant brain tumor in adults. Under the 2021 WHO criteria, the diagnosis of GBM is strictly reserved for diffuse astrocytic tumors that are IDH-wildtype and H3-wildtype [71].

Beyond the defining absence of IDH mutations, these tumors are characterized by specific molecular hallmarks, including TERT promoter mutations, EGFR amplification, and the combined gain of chromosome 7 with loss of chromosome 10 (+ 7/−10), which collectively drive aggressive proliferation and genomic instability [72]. Regarding their cellular origin, although GBM cells express astrocytic markers, lineage-tracing studies suggest they often originate from neural stem cells in the subventricular zone (SVZ) or from oligodendrocyte precursor cells that undergo dedifferentiation to acquire an astrocyte-like, stem-like state [73, 74]. In terms of clinical behavior, these tumors typically arise de novo in older adults (median age ~ 60 years), exhibit rapid progression with a median survival of approximately 15 months, and inevitably recur due to diffuse infiltration into the brain parenchyma [75, 76].

#### Astrocytoma, IDH-mutant

The term “secondary glioblastoma” is now largely obsolete. Tumors previously classified as such—those arising from lower-grade precursors harboring IDH1 or IDH2 mutations—are now designated as Astrocytoma, IDH-mutant [77]. The diagnosis of astrocytoma, distinct from oligodendroglioma, is now defined by a specific molecular signature requiring an IDH1 or IDH2 mutation alongside the loss of nuclear ATRX expression and/or TP53 mutation. The loss of ATRX, a chromatin remodeler, leads to the alternative lengthening of telomeres (ALT) pathway, a feature that strictly demarcates the astrocytoma lineage from 1p/19q-codeleted oligodendrogliomas [78, 79]. A critical consequence of the IDH mutation is the production of the oncometabolite D-2-hydroxyglutarate (2-HG). This molecule is secreted into the microenvironment and taken up by surrounding wild-type astrocytes, inducing a unique, epigenetically repressed phenotype distinct from the reactive state seen in IDH-wildtype GBM [80]. Clinically, IDH-mutant Grade 4 Astrocytomas carry a significantly better prognosis than IDH-wildtype GBM, partly because these “educated” astrocytes in the TME exhibit a less inflammatory and invasive profile [40].

### Role of astrocytes in the tumor microenvironment

**Fig. 2:**
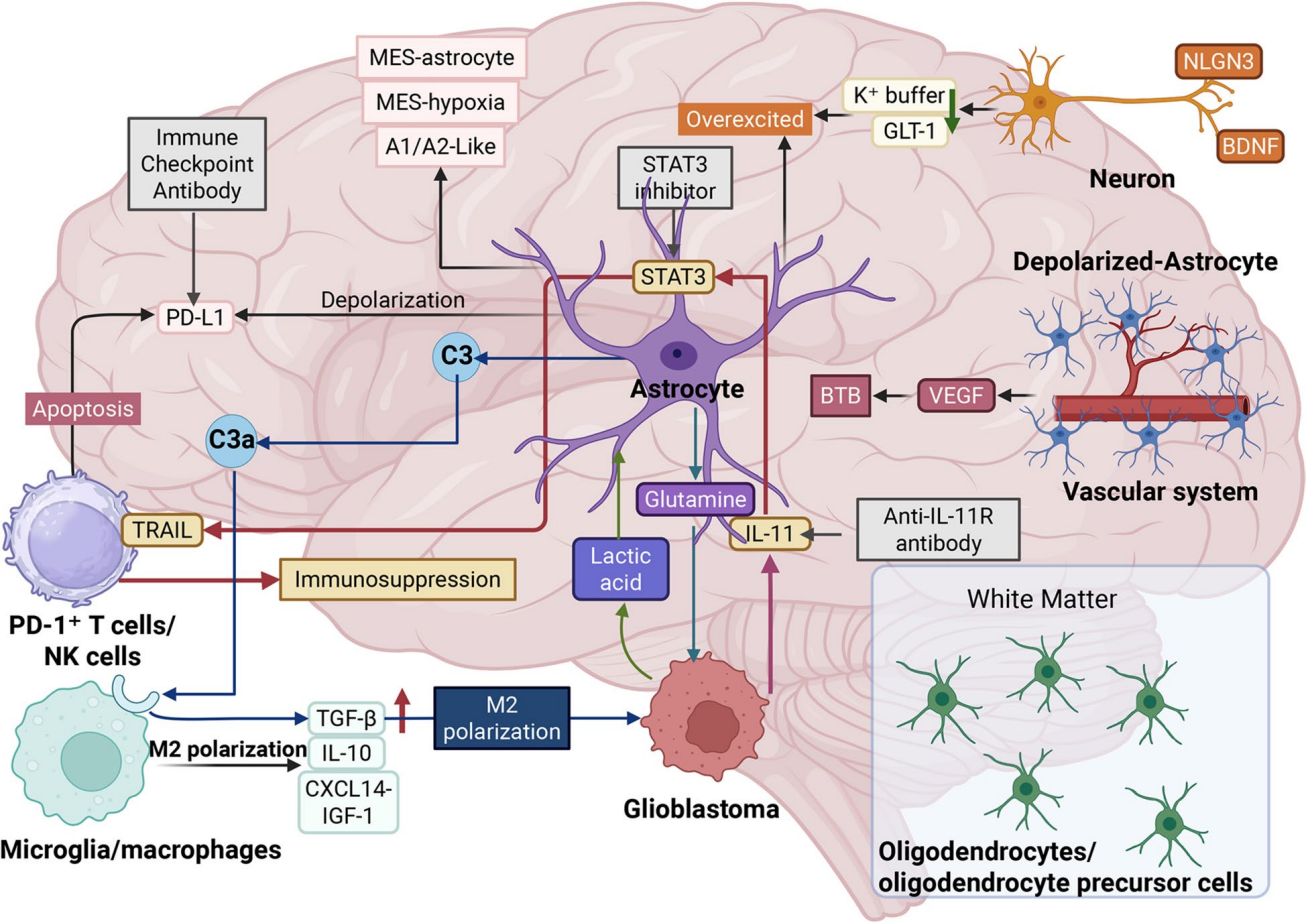
The multifaceted role of tumor-associated astrocytes (TAAs) in the glioblastoma microenvironment. Astrocytes engage in complex crosstalk with various cellular components within the tumor microenvironment (TME). They suppress antitumor immunity by expressing programmed death-ligand 1 (PD-L1) and, upon stimulation by tumor-secreted interleukin-11 (IL-11), by expressing TNF-related apoptosis-inducing ligand (TRAIL) to induce T-cell apoptosis. TAAs also foster an immunosuppressive milieu by releasing factors such as transforming growth factor-beta (TGF-β) and interleukin-10 (IL-10), which drive the polarization of microglia and macrophages toward a protumorigenic M2 phenotype. Furthermore, astrocytes provide metabolic support to glioblastoma cells by supplying glutamine and lactate and promote angiogenesis and blood–tumor barrier (BTB) disruption via vascular endothelial growth factor (VEGF) secretion. Their interactions with neurons, modulated by factors including neuroligin-3 (NLGN3) and brain-derived neurotrophic factor (BDNF), can induce neuronal hyperexcitability while simultaneously promoting tumor growth. The figure also illustrates how distinct TAA states (e.g., mesenchymal [MES] astrocytes, MES-hypoxia) and key signaling pathways (e.g., STAT3, complement C3/C3a receptor) contribute to this network, suggesting potential therapeutic interventions such as STAT3 inhibitors and immune checkpoint blockade. This figure was created with BioRender.com. Abbreviations: TAA, tumor-associated astrocyte; TME, tumor microenvironment; PD-L1, programmed death-ligand 1; IL-11, interleukin-11; TRAIL, TNF-related apoptosis-inducing ligand; TGF-β, transforming growth factor-beta; IL-10, interleukin-10; BTB, blood-tumor barrier; VEGF, vascular endothelial growth factor; NLGN3, neuroligin-3; BDNF, brain-derived neurotrophic factor; MES, mesenchymal; STAT3, signal transducer and activator of transcription 3; NK, natural killer; GLT-1, glutamate transporter-1

The TME of brain tumors is a unique niche shaped by the brain's specialized anatomy. Unlike peripheral tumors where fibroblasts dominate the stroma, astrocytes are the principal stromal component in the brain, accounting for up to 50% of the total cellular mass in the tumor bulk [81–83]. In this tumor ecosystem, TAAs are not static but exhibit significant spatial heterogeneity (Fig. 2, Table 1).

**Table 1:** Key interactions of astrocytes and functional consequences of their heterogeneity within the tumor microenvironment

Interaction partners	Key Astrocyte-Mediated Processes	Consequences of Astrocyte Heterogeneity	Reference
Microglia	Inflammatory regulation (A1 induction), immune cell recruitment (CCL2, CXCL10), polarization (TGF-β/ORM2 → M2/Reparative)	Distinct TAA subtypes create unique immune milieus: A1-inducing (neurotoxic) vs. M2-promoting (immunosuppressive/pro-tumor)	[51, 55, 58, 84]
Neurons	Glutamate clearance (EAATs), K⁺ buffering (Kir4.1), neurovascular coupling, metabolic supply (lactate)	Loss of homeostatic subtypes leads to excitotoxicity and seizures; maintained metabolic coupling supports tumor-associated neuronal activity	[7, 9, 12, 16, 85]
Oligodendrocytes	Myelination support, lipid metabolism	A1 astrocytes kill oligodendrocytes (demyelination); mesenchymal TAAs may facilitate invasion along white matter tracts	[19, 20, 22, 45, 86]
Tumor cells (GBM)	Gap junction coupling (Cx43), mitochondrial transfer (TNTs), ECM remodeling (MMPs)	Forms a “syncytium of resistance” (calcium buffering, drug resilience); provides direct physical and metabolic support for invasion	[36, 87–91]
Vascular system	BBB maintenance (endfeet/AQP4), angiogenesis (VEGF), immune cell trafficking	Perivascular astrocyte uncoupling leads to BBB breakdown (BTB); hypoxic astrocytes drive disorganized angiogenesis	[3, 92–94]

#### The "Stiff" Tumor: mechanotransduction and matrix stiffness

Emerging research highlights that the physical properties of the TME are as crucial as chemical signals. GBM tissues are significantly stiffer than normal brain parenchyma. Reactive astrocytes are key contributors to this stiffening through the secretion of matrix proteins such as tenascin-C, fibronectin, and hyaluronan [95–97]. Astrocytes sense this increased stiffness via mechanosensitive channels (e.g., Piezo1) and integrin receptors. Activation of these sensors triggers nuclear translocation of the transcriptional co-activators YAP/TAZ, which further amplifies a pro-tumorigenic, mesenchymal phenotype in astrocytes. This creates a feed-forward loop: tumor stiffening activates astrocytes → activated astrocytes secrete more ECM → the tumor becomes stiffer and more aggressive [98–100].

#### Spatial heterogeneity: distinct functional niches

TAA function is strongly dictated by their spatial location relative to the tumor mass. In the tumor core (perinecrotic niche), where conditions are hypoxic, astrocytes are often sparse or degenerated. The surviving population, termed “MES-hypoxia” astrocytes, is metabolically adapted to glycolysis and serves as a primary source of VEGF, driving the disorganized angiogenesis characteristic of GBM [101, 102]. In the perivascular niche, perivascular astrocytes (PVAs) are critical for blood-tumor barrier (BTB) maintenance. In high-grade tumors, PVAs undergo pathological retraction of their endfeet from vessel walls and lose polarized aquaporin-4 (AQP4) expression, leading to “vascular decoupling” and BBB breakdown [92, 103, 104]. Finally, at the invasive margin (peritumoral zone), reactive astrocytes initially form a “glially limiting” border aimed at containing the tumor. However, GBM-secreted factors eventually “educate” these astrocytes, converting them from a containment barrier into a scaffold that facilitates diffuse infiltration of single tumor cells along white matter tracts [87, 105, 106].

### Impact of astrocytes on tumor invasion, proliferation, and metastasis

The interplay between malignant cells and TAAs drives the hallmark features of brain cancer. This is not a passive support system but an active, bidirectional collaboration that remodels the brain's physical and metabolic landscape [107].

#### Driving invasion: the ECM and connexin connection

GBM is notoriously invasive, and TAAs facilitate this infiltration through several molecular mechanisms. First, reactive astrocytes are prolific secretors of enzymes involved in matrix remodeling, particularly MMP-2 and MMP-9. These enzymes degrade extracellular matrix (ECM) components, effectively clearing “migration highways” for tumor cell infiltration [108]. Additionally, astrocytic tenascin-C (TNC) upregulates MMP-12 in glioma cells, further amplifying invasive capability [90]. Second, direct physical contact is established via connexin-43 (Cx43) gap junctions. This coupling enables the bidirectional transfer of signaling molecules, such as invasion-promoting microRNAs (e.g., miR-19b), from GBM cells to astrocytes [88, 109]. Third, under tumor-derived signals, astrocytes can undergo a mesenchymal transition, expressing high levels of vimentin and TNC. This state supports the tumor's “leading edge,” physically guiding malignant cells along white matter tracts [86, 89, 110]. Recent findings also highlight the role of GlialCAM, which is heterogeneously expressed in GBM. High GlialCAM expression promotes cell–cell adhesion and proliferation in the tumor core, whereas low expression is associated with enhanced parenchymal and vascular invasion [19].

#### Fueling proliferation: metabolic and organelle support

Astrocytes act as metabolic hubs, a function co-opted in the TME to fuel rapid tumor growth. In a striking example of organelle hijacking, GBM cells form Tunneling Nanotubes (TNTs)—thin membrane bridges—with neighboring astrocytes. Through these conduits, astrocytes transfer functional mitochondria to GBM cells, boosting the tumor's oxidative phosphorylation capacity and conferring resistance to metabolic stress, a process often upregulated by oxidative stress and chemotherapy [35, 36]. Beyond organelle transfer, TAAs support tumor metabolism through metabolic coupling. Astrocytes upregulate glutamine transporters, such as alanine-serine-cysteine transporter 2(ASCT2) to serve as a nitrogen source for tumor nucleotide synthesis [111]. In hypoxic regions, a “lactate shuttle” is established where astrocytes export lactate via monocarboxylate transporters 1 and 4(MCT1/4) transporters to support tumor ATP production via a reverse Warburg effect [112]. Recent studies further reveal that neuronal activity itself promotes glioma growth; activity-induced, ADAM10-mediated shedding of neuroligin-3 (NLGN3), in conjunction with BDNF, acts as a protumorigenic factor driving glioma cell proliferation via AMPA receptor-mediated currents [113].

#### The paradox of Brain Metastasis (BrM)

For secondary brain tumors (metastases from lung, breast, or melanoma), the role of astrocytes evolves over time, presenting a paradox. Upon initial metastatic seeding, reactive astrocytes act defensively; they surround micrometastases and release plasminogen activators (PAs) to generate plasmin, inducing cancer cell apoptosis. This phase represents a major bottleneck for colonization [114]. However, surviving cancer cells eventually reprogram astrocytes to a supportive, pro-metastatic state. These co-opted astrocytes downregulate PA inhibitors (e.g., TIMP-1) and secrete survival factors such as IL-6 and BDNF, protecting metastatic seeds from chemotherapy [51, 115]. Specific mechanisms have been identified; for instance, in lung cancer brain metastasis, astrocytes secrete Wnt5a. This promotes mGluR1 expression in cancer cells, where mGluR1 physically interacts with and stabilizes EGFR, enhancing downstream ERK signaling and thereby promoting cancer cell proliferation and survival [116].

### Contribution of astrocytes to immune evasion

One of the most critical roles of TAAs is fostering an immunologically cold microenvironment. TAAs actively reinforce the brain's immune privilege through specific molecular checkpoints and cytokine networks [117].

#### The IL-11/TRAIL apoptosis axis

A pivotal mechanism of astrocyte-mediated immunosuppression involves active T-cell elimination. GBM cells secrete interleukin-11 (IL-11), which binds to receptors on astrocytes, activating the STAT3 pathway. STAT3 activation induces astrocytic expression of TNF-related apoptosis-inducing ligand (TRAIL). This astrocytic TRAIL binds to death receptors, such as death receptor 5 (DR5), on infiltrating T cells, triggering their apoptosis. This mechanism effectively creates a “kill zone” around the tumor, eliminating anti-tumor immune cells [46].

#### PD-L1 and checkpoint inhibition

TAAs are a major source of programmed death-ligand 1 (PD-L1) in the TME. Driven by signals such as interferon-β and IL-10, astrocytes upregulate surface PD-L1 expression. Engagement of PD-1 on infiltrating cytotoxic T lymphocytes (CTLs) by PD-L1 induces T-cell exhaustion and anergy [118]. The combined expression of PD-L1 and TRAIL on the astrocytic network presents a formidable barrier to immunotherapy.

#### Cytokine orchestration and macrophage polarization

TAAs sustain an immunosuppressive milieu through the secretion of TGF-β and IL-10. This cytokine TGF-β acts as a master regulator, inhibiting natural killer (NK) cell activity and blocking CTL proliferation [118]. Astrocytes also secrete chemokines like CCL2 and CSF1 to recruit monocytes from the periphery. Within the TME, astrocyte-derived TGF-β and IL-10 polarize these cells into M2-like tumor-associated macrophages (TAMs), which further suppress immune responses and support angiogenesis [51, 63].

## Cross-talk between neuroinflammation and brain cancer via astrocytes

The relationship between neuroinflammation and brain cancer is not merely correlative but causally intertwined, with astrocytes serving as the central signaling hub facilitating this cross-talk. Within the TME, the distinction between a “wound healing” response and a “tumor-promoting” pathology becomes blurred. Malignant cells hijack conserved inflammatory pathways—including NF-κB, STAT3, and the emerging cyclic GMP-AMP synthase-stimulator of interferon genes (cGAS-STING) axis—to reprogram astrocytes, converting them from defenders of neural homeostasis into active drivers of oncogenesis. This section synthesizes the advanced molecular mechanisms through which inflammatory signaling translates into tumor progression, immune evasion, and therapeutic resistance.

### Epigenetic and hypoxic reprogramming of astrocytes

**Fig. 3:**
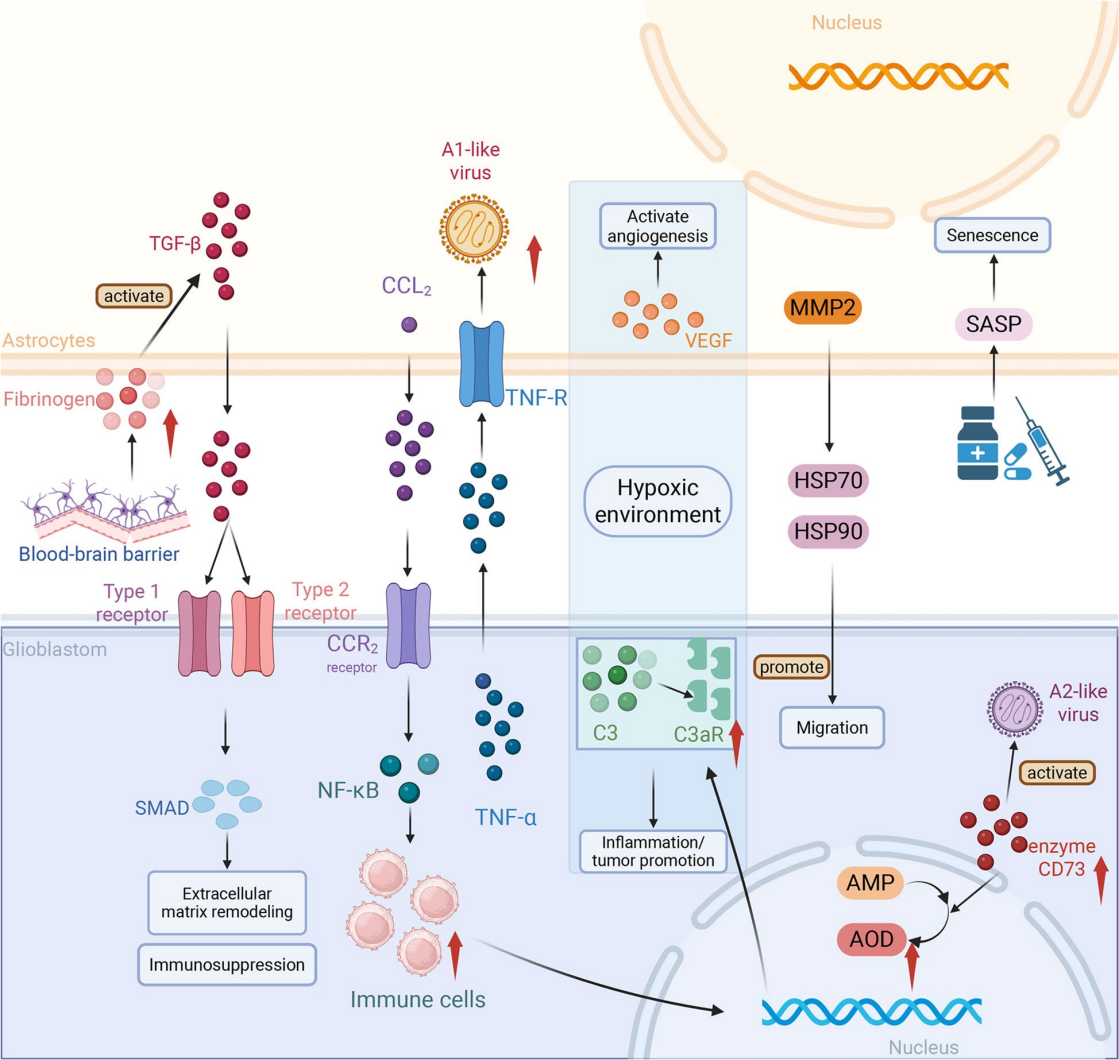
Main drivers of astrocyte heterogeneity in the brain tumor microenvironment. Signals from the tumor and its surroundings actively reprogram astrocyte function through multiple mechanisms. A compromised blood–brain barrier allows the influx of blood-derived factors such as fibrinogen, which, alongside tumor-derived TGF-β, activates SMAD signaling to drive extracellular matrix remodeling and immunosuppression. Concurrently, inflammatory signaling plays a major role; tumor-secreted CCL2 recruits peripheral immune cells, while microglia-derived TNF-α triggers a pro-inflammatory astrocytic state. In the tumor core, hypoxic conditions stabilize HIF-1α, stimulating astrocytes to secrete VEGF for angiogenesis and upregulate complement component C3, which enhances inflammation and tumor cell migration via the C3a receptor. Finally, therapeutic interventions such as radiation or chemotherapy can induce a senescence-associated secretory phenotype (SASP), resulting in the chronic release of factors that promote therapeutic resistance. This figure was created with BioRender.com. Abbreviations: TGF-β, transforming growth factor-beta; SMAD, small mothers against decapentaplegic; CCL2, C–C motif chemokine ligand 2; TNF-α, tumor necrosis factor-alpha; TNF-R, tumor necrosis factor receptor; NF-κB, nuclear factor-kappa B; HIF-1α, hypoxia-inducible factor-1 alpha; VEGF, vascular endothelial growth factor; C3, complement component 3; C3aR, C3a receptor; MMP2, matrix metalloproteinase-2; HSP70, heat shock protein 70; HSP90, heat shock protein 90; CD73, cluster of differentiation 73; SASP, senescence-associated secretory phenotype

Astrocyte activation in the TME is a multi-step process driven by paracrine signals, metabolic stressors, and systemic physiological states. Unlike the acute astrogliosis seen in stroke, TAA activation involves profound epigenetic remodeling and metabolic rewiring that locks them into a chronic, tumor-supportive state (Fig. 3, Table 2).

**Table 2:** Key subtypes/states of tumor-associated astrocytes (TAAs): markers, functions, and drivers

TAA Subtype/State	Key Markers	Putative Function	Key Drivers/Context	Reference
Classical reactive/scar-forming	GFAP↑, Vimentin↑, STAT3-p↑	Physical Barrier (Containment), Restriction of inflammation spread	JAK/STAT3 pathway, Acute injury, Tumor border (early)	[31, 33]
A1-like (Neurotoxic)	C3, Complement factors	Neuronal and oligodendrocyte killing, loss of synaptic support	Microglia-derived IL-1α, TNF-α, C1q	[22, 45]
A2-like/Reparative	Neurotrophic factors (BDNF, VEGF)	Neuronal survival, Tissue repair, BBB maintenance	Ischemia, Repair signals	[22]
Metabolic supportive	ASCT2↑, MCT1/4↑, Glutamine Synthetase changes	Supplies glutamine/lactate ("Reverse Warburg"); mitochondrial donation	Tumor metabolic demand, hypoxia, metabolic parasitism	[14, 15, 112, 119]
Pro-invasive/Mesenchymal	Vimentin↑, TNC↑, MMP-2/9↑, Cx43↑	ECM Degradation, "Leading edge" guidance, Invasion support	TGF-β, Tumor contact, Stiffness (Mechanotransduction)	[86, 90, 109, 110]
Immunosuppressive	PD-L1↑, TRAIL↑, TGF-β↑, IL-10↑	T-cell apoptosis (TRAIL), T-cell exhaustion (PD-L1), M2 macrophage recruitment	IL-11/STAT3 axis, IFN-β, Chronic inflammation	[46, 51, 55, 56, 63, 118]
Hypoxia-related (MES-hypoxia)	HIF-1α↑, VEGF↑, PKM2↑	Angiogenesis, Glycolytic switch, MDSC recruitment	Hypoxia (Tumor core), Necrosis	[56, 103, 120, 121]
SASP (senescence-associated secretory phenotype)	p16INK4a↑, p21↑, IL-6↑, IL-8↑	Pro-inflammatory "secretome," Promotes GSC stemness and recurrence	Radiotherapy, Chemotherapy (DNA damage)	[37, 42, 43]

#### The "Hijacked Repair" mechanism: NF-κB and STAT3

Tumor cells secrete factors that mimic injury signals, effectively acting as "wounds that never heal." Cytokines such as IL-1β and TNF-α, secreted by microglia and tumor cells, trigger NF-κB activation in astrocytes. Unlike in neurodegeneration, this activation within the TME promotes a “mixed” phenotype where astrocytes support tissue remodeling via MMPs while simultaneously suppressing adaptive immunity [122]. Concurrently, the JAK/STAT3 pathway functions as a master regulator and is constitutively activated in TAAs. Phosphorylated STAT3 drives the expression of structural proteins like vimentin and GFAP, characteristic of reactive astrogliosis, and critically upregulates PD-L1 and TRAIL expression, thereby linking structural reactivity to immune suppression [46, 123].

#### Epigenetic reprogramming: locking in the phenotype

Emerging evidence indicates that TAA transformation is underpinned by stable epigenetic changes, ensuring astrocytes remain “primed” to support cancer even if tumor-derived signals are temporarily removed [124]. For instance, the histone methyltransferase enhancer of zeste homolog 2 (EZH2) is frequently upregulated in TAAs, catalyzing the trimethylation of histone H3 at lysine 27 (H3K27me3). This repressive mark silences tumor-suppressor genes and differentiation factors, effectively preventing TAAs from reverting to a quiescent state [125, 126]. Similarly, in IDH-mutant astrocytomas, the secretion of the oncometabolite D-2-hydroxyglutarate (D-2-HG) competitively inhibits ten-eleven translocation(TET) family DNA demethylases. This leads to genome-wide DNA hypermethylation in adjacent astrocytes, creating a permissive niche tailored for slower tumor growth [126–128].

#### Hypoxia-driven reprogramming

As the tumor mass outgrows its vascular supply, necrotic cores generate profound hypoxia, acting as a potent environmental switch. Hypoxia stabilizes hypoxia-inducible factor-1α (HIF-1α) in astrocytes, which orchestrates a multifaceted response. HIF-1α upregulates VEGF to drive angiogenesis and simultaneously triggers a metabolic shift toward aerobic glycolysis (the Warburg effect) by increasing the expression of glycolytic enzymes such as PKM2 and LDHA [121, 129, 130]. Furthermore, hypoxic astrocytes secrete specific ECM proteins, including tenascin-C and periostin, which stiffen the TME and facilitate macrophage recruitment. This resulting “MES-hypoxia” state is functionally distinct from normoxic reactive astrocytes and represents a critical node of drug resistance [120, 131, 132].

#### Senescence-Associated Secretory Phenotype (SASP)

Therapeutic interventions, particularly radiotherapy and alkylating chemotherapy (e.g., temozolomide), can inadvertently induce a senescent state in astrocytes. DNA damage triggers cell cycle arrest via p16INK4a and p21CIP1. These non-proliferating astrocytes acquire a SASP. SASP astrocytes become chronic “inflammatory factories,” secreting high levels of IL-6, IL-8, and hepatocyte growth factor (HGF). Paradoxically, this secretome protects surviving tumor cells from apoptosis and promotes invasion, contributing to the near-inevitability of GBM recurrence [41–43].

#### Systemic drivers: the gut-brain axis

Emerging evidence suggests that systemic cues, particularly from the gut-brain axis, also modulate TAA reactivity. Gut microbiota metabolize dietary tryptophan into indoles and other ligands that can cross the BBB and activate the aryl hydrocarbon receptor (AhR) on astrocytes. Under physiological conditions, AhR signaling limits CNS inflammation. However, in glioma patients with gut dysbiosis, altered metabolite levels may lead to insufficient AhR activation, potentially unleashing a pro-inflammatory TAA phenotype that supports tumor growth [37–39].

#### Hormonal signaling

Sex hormones and locally synthesized neurosteroids constitute a critical yet underappreciated class of regulators driving TAA heterogeneity. Astrocytes express key steroidogenic enzymes such as aromatase, enabling de novo steroid hormone production that creates a distinct neuroendocrine milieu. For instance, reactive astrocytes surrounding brain metastases upregulate aromatase, converting adrenal-derived precursors into estrogens. These estrogens can enhance metastatic growth—even in ER-negative primary tumors—by acting on ER + astrocytes to induce the secretion of supportive factors like BDNF [40]. Additionally, hormones shape the brain’s immune landscape; estrogen, for example, can mobilize and activate immunosuppressive myeloid-derived suppressor cells (MDSCs) and regulatory T cells (Tregs), potentially fostering an immune-evasive TME [40].

#### Cholinergic signaling

The cholinergic system, pivotal for neurotransmission, is notably hijacked by certain tumors, such as lung adenocarcinoma (LUAD), which is characterized by elevated acetylcholine production. Tumor-derived acetylcholinesterase (AChE) is particularly implicated in facilitating brain metastasis by targeting astrocytes. High AChE levels induce astrocyte apoptosis by overactivating astrocytic cholinergic receptors, leading to excessive Ca2 + influx and ATP release. This targeted destruction of astrocytes disrupts the BBB, a critical step for metastatic colonization [39]. Furthermore, high AChE levels impair CD8 + T lymphocyte function and promote their apoptosis, contributing to an immune-suppressive niche.

### Hijacking metabolic homeostasis: the "Reverse Warburg" effect

Astrocytes, as natural metabolic hubs of the brain, are forced into a state of “metabolic slavery” by GBM cells, fueling rapid tumor growth and radioresistance.

#### The IL-6/STAT3 feed-forward loop

Interleukin-6 (IL-6) is a cornerstone of the pro-tumorigenic microenvironment. Triggered by tumor-derived IL-1β, astrocytes secrete abundant IL-6, which acts in an autocrine manner to sustain astrocytic STAT3 activation and in a paracrine manner on glioma cell gp130 receptors [133, 134]. In tumor cells, this IL-6 signaling promotes survival, stemness via Notch pathway crosstalk, and invasion. Clinically, high cerebrospinal fluid IL-6 levels correlate with poor prognosis and resistance to bevacizumab [135].

#### The cGAS-STING pathway: a double-edged sword

The innate immune sensing of cytosolic DNA via the cGAS-STING pathway represents a cutting-edge area of research with dual roles in the TME. Activation typically occurs when genomic instability in GBM cells leads to cytoplasmic double-stranded DNA (dsDNA) or micronuclei, which can be transferred to astrocytes via gap junctions or exosomes. Astrocytes may also sense mitochondrial DNA (mtDNA) released from dying cells [136]. While cytosolic DNA activates cGAS to produce 2′3′-cGAMP and subsequently activate STING to ostensibly induce anti-tumor type I interferons, the outcome in the chronic GBM TME is different. Prolonged STING signaling in astrocytes can drive a non-canonical, NF-κB-dependent inflammatory program. This chronic inflammation fosters an immunosuppressive niche characterized by MDSC recruitment rather than the expected cytotoxic T-cell infiltration [137–139].

#### TGF-β: the immunosuppressive blanket

Transforming growth factor-beta (TGF-β), predominantly the TGF-β2 isoform derived from astrocytes, serves as the most potent immunosuppressive cytokine in the TME. It acts on multiple fronts to stifle immune surveillance, inhibiting T-cell proliferation, blocking NK cell cytotoxicity, and driving microglial polarization toward an M2-like phenotype. Crucially, astrocytic TGF-β also exerts vascular effects by downregulating endothelial adhesion molecules (ICAM-1/VCAM-1). This prevents immune effector cell extravasation into the tumor parenchyma, effectively sealing the tumor from immune attack [55, 140].

### Astrocyte-tumor cell interactions: the syncytium of resistance

The cross-talk between astrocytes and tumor cells extends to direct physical coupling and complex metabolic exchange, integrating the tumor and stroma into a singular, resistant functional unit [141].

#### Gap junctions: the electrochemical syncytium

Cx43-mediated gap junctions (GJs) create a cytosolic continuum between TAAs and glioma cells. This coupling provides chemo-protection via calcium buffering; when tumor cells face cytotoxic stress and experience a lethal surge in intracellular Ca2 +, connected astrocytes act as a massive “calcium sink,” absorbing excess Ca2 + and preventing tumor cell apoptosis [142]. Furthermore, this syncytium facilitates cGAMP transfer and immune evasion. While cGAMP is typically produced in tumor cells to trigger an interferon response via the cGAS-STING pathway, tumor cells shuttle cGAMP into astrocytes via Cx43 channels. This "dilutes" the danger signal within the tumor cell, preventing intrinsic immune activation, while simultaneously triggering a non-canonical inflammatory response in the astrocytes that further suppresses T-cell function [138, 143].

#### Tunneling Nanotubes (TNTs): organelle and information hijacking

Distinct from gap junctions, Tunneling Nanotubes (TNTs) are long, actin-based membrane protrusions that enable the unidirectional transfer of large cellular cargoes. Under conditions of metabolic stress or radiation injury, GBM cells extend TNTs to neighboring astrocytes to facilitate mitochondrial donation. Through these conduits, astrocytes transfer healthy, functional mitochondria to respiratory-impaired tumor cells, restoring oxidative phosphorylation, reducing reactive oxygen species (ROS), and conferring acute therapy resistance [91, 143]. Additionally, TNTs facilitate the transfer of genetic information; for example, resistance to temozolomide (TMZ) can be acquired by MGMT-negative tumor cells through the receipt of MGMT mRNA from astrocytes via TNTs and extracellular vesicles, enabling survival without genomic alteration [91, 144].

#### Lactate metabolic coupling

Driven by the “reverse Warburg effect,” astrocytes are reprogrammed to act as metabolic feeders for glioblastoma. They upregulate aerobic glycolysis and export lactate via MCT1/4 transporters. Tumor cells subsequently import this lactate to fuel their TCA cycle and nucleotide synthesis, thereby sparing their own glucose for other biosynthetic pathways and sustaining rapid proliferation [145–147].

### Immune cell recruitment and modulation by astrocytes

Beyond direct T-cell suppression, astrocytes orchestrate the broader immune landscape by recruiting suppressive myeloid cells and constructing physical barriers.

#### Recruitment of suppressive myeloid cells

Astrocytes serve as a central chemokine hub within the TME, dictating immune cell infiltration to favor suppression. As the primary source of CCL2 (MCP-1), TAAs establish a gradient that recruits CCR2 + inflammatory monocytes from the periphery. Once within the TME, astrocyte-derived factors such as GM-CSF and TGF-β induce these cells to differentiate into M2-like, immunosuppressive tumor-associated macrophages (TAMs) [148, 149]. Additionally, hypoxic astrocytes secrete CXCL12 (SDF-1) and macrophage migration inhibitory factor (MIF), which specifically recruit myeloid-derived suppressor cells (MDSCs)—immature myeloid cells that potently inhibit T-cell activation and promote angiogenesis [149, 150].

#### Microglial polarization and reprogramming

Astrocytes and microglia engage in reciprocal regulation that evolves with tumor progression. While microglia can initially induce the neurotoxic A1 astrocyte phenotype, the dynamic shifts in the chronic TME. Astrocytes secrete factors like orosomucoid-2 (ORM2) and osteopontin (OPN), which dampen pro-inflammatory (M1-like) microglial activation and actively skew them toward a reparative, pro-tumor state [151, 152].

#### Physical exclusion: the "Immune Desert"

Beyond signaling, astrocytes contribute to immune evasion through physical restructuring of the TME. Reactive astrocytes secrete a dense, cross-linked ECM rich in hyaluronan and chondroitin sulfate proteoglycans. This stiff matrix acts as a physical barrier that restricts cytotoxic T-cell motility, trapping them in perivascular spaces and preventing their infiltration into the tumor core. This phenomenon of “immune exclusion” creates an immunological desert and is a primary reason for the failure of checkpoint inhibitors in GBM [153–155].

### Functional plasticity of astrocytes in inflammatory and tumor environments

**Fig. 4:**
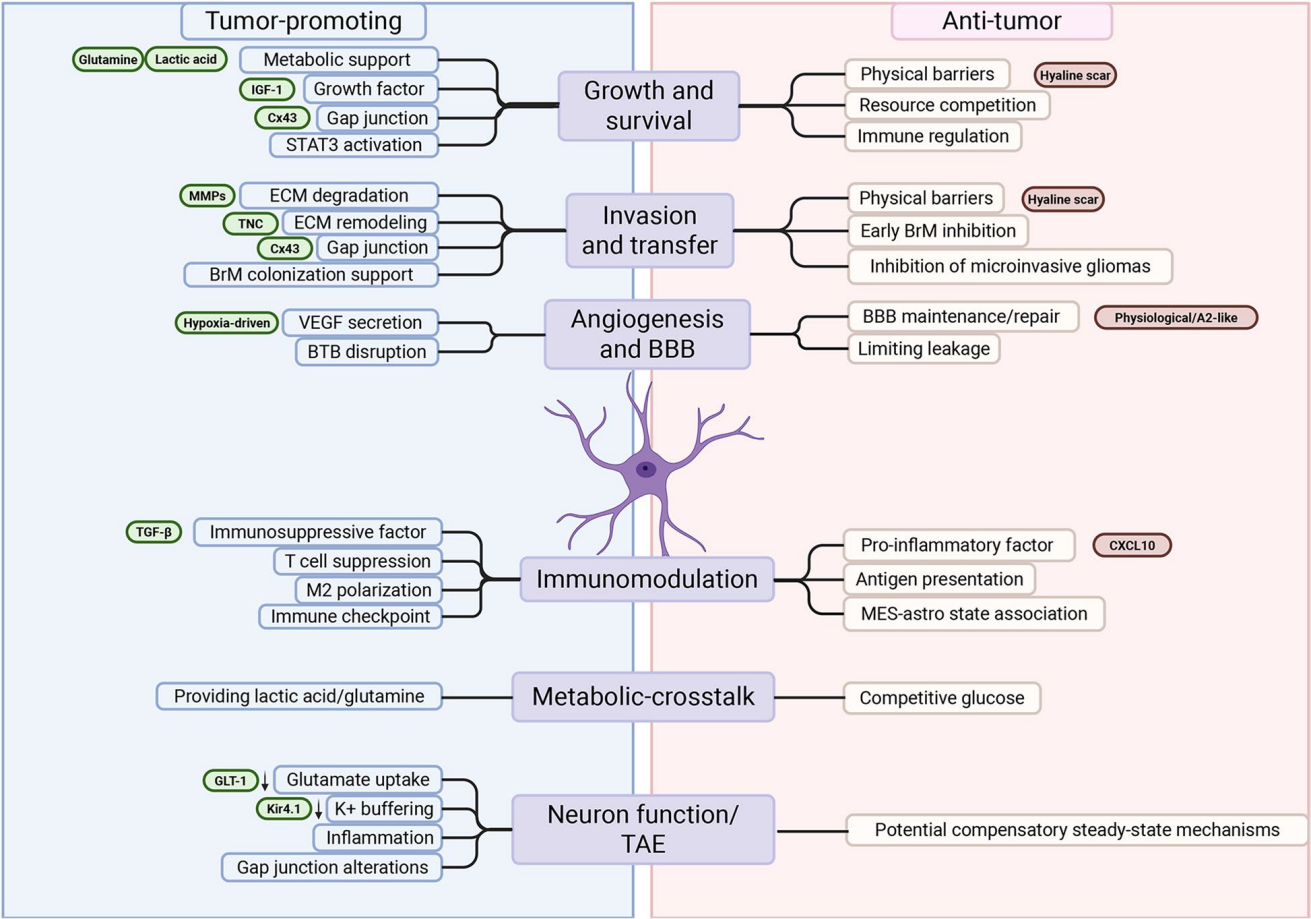
Context-dependent dual roles of reactive astrocytes in brain tumor biology. The net effect of tumor-associated astrocyte (TAA) activity critically depends on the specific molecular and spatial context of the tumor microenvironment. Regarding growth and survival, TAAs provide metabolic support through lactate and glutamine transport and activate STAT3 pathways, yet they can simultaneously restrict tumor expansion through glial scar formation and resource competition. Similarly, in the context of invasion and metastasis, astrocytes promote dissemination by degrading the extracellular matrix via MMPs and depositing tenascin-C, while anisomorphic scarring acts as a physical barrier to containment. Vascular interactions are equally dualistic; TAAs may disrupt the blood–brain barrier through VEGF-driven angiogenesis or conversely maintain barrier integrity and vascular tone. Finally, immunomodulation varies significantly, with astrocytes either fostering immunosuppression via TGF-β secretion or driving pro-inflammatory responses and antigen presentation as seen in the mesenchymal state. This figure was created with BioRender.com. Abbreviations: TAA, tumor-associated astrocyte; STAT3, signal transducer and activator of transcription 3; MMPs, matrix metalloproteinases; TNC, tenascin-C; BBB, blood–brain barrier; BTB, blood-tumor barrier; VEGF, vascular endothelial growth factor; TGF-β, transforming growth factor-beta; MES, mesenchymal; BrM, brain metastasis; Cx43, connexin-43; IGF-1, insulin-like growth factor-1; GLT-1, glutamate transporter-1; Kir4.1, inwardly rectifying potassium channel 4.1; ECM, extracellular matrix

The most defining—and clinically challenging—feature of TAAs is their plasticity: the ability to dynamically switch functions based on the evolving inflammatory context and therapeutic pressure. Astrocytes are not fixed in a “good” or “bad” state but are entrained by the TME (Fig. 4).

#### The "Switch" from containment to support: anisomorphic vs. isomorphic astrogliosis

In brain injury and glioma, astrogliosis manifests in two forms that are mirrored and manipulated during progression. During early tumorigenesis, astrocytes undergo anisomorphic astrogliosis, forming a dense, overlapping meshwork with strong cell–cell junctions. This structure acts as a physical “glial scar” intended to wall off the lesion and contain inflammation [156–158]. However, as the tumor evolves, accumulating cytokines like TGF-β and IL-6 overwhelm this defense, leading to late co-option and isomorphic astrogliosis. In this phase, astrocytes dissolve rigid cell–cell junctions and align their processes parallel to white matter tracts, providing “anisotropic” physical guidance cues that actively lead tumor cells deep into the parenchyma [157, 159].

#### Therapy-induced plasticity: the dark side of treatment

Standard interventions can inadvertently trigger maladaptive astrocyte states that fuel recurrence. For instance, ionizing radiation induces double-strand DNA breaks in astrocytes, driving many into a state of permanent cell cycle arrest known as senescence. These senescent astrocytes acquire a SASP driven by the p21/NF-κB axis and chronically secrete pro-inflammatory cytokines (IL-6, IL-8), growth factors (HGF), and proteases. This inflamed milieu paradoxically supports glioma stem cell (GSC) survival, protects them from further radiation, and primes the niche for rapid recurrence [43, 160]. Similarly, treatment with the alkylating agent TMZ upregulates transglutaminase 2 (TGM2) in reactive astrocytes. TGM2 crosslinks ECM proteins such as fibronectin, significantly increasing TME stiffness. This stiff niche activates mechanotransduction pathways (YAP/TAZ) in GSCs to enhance their stemness and chemoresistance, effectively creating a physical barrier that protects the tumor [161]. Furthermore, longitudinal studies of patient-paired primary versus recurrent GBM samples reveal that recurrent tumors often shift to a mesenchymal phenotype. This transition is partly driven by AP-1 signaling in astrocytes, highlighting how TAA plasticity evolves under therapeutic pressure to drive recurrence [162].

#### Spatial plasticity: core vs. margin

TAA phenotype is further dictated by intratumoral geography. In the hypoxic or necrotic tumor core, astrocytes are often senescent or degraded. The surviving “MES-hypoxia” population is metabolically adapted to glycolysis and is dedicated to angiogenesis (VEGF secretion) and immune exclusion (MDSC recruitment) [163]. Conversely, astrocytes at the invasive margin (peritumoral zone) are typically hypertrophic and viable. They interact directly with infiltrating glioma cells and are characterized by high connexin-43 expression for metabolic coupling and high MMP secretion for ECM degradation [164, 165]. This spatial compartmentalization implies that a single therapeutic agent may not target all TAAs effectively, suggesting that margin-targeting strategies (e.g., gap junction blockers) may need to be combined with core-targeting approaches (e.g., anti-angiogenics).

## Therapeutic strategies targeting astrocytes

The revelation that astrocytes act as central architects of the glioblastoma ecosystem—driving invasion, metabolic fueling, and immune exclusion—demands a paradigm shift in therapeutic strategy. Traditional “tumor-centric” approaches focused on killing rapidly dividing cancer cells have largely failed to yield durable cures, in part because they spare the supportive astrocytic stroma [166, 167]. To break the cycle of recurrence, future therapies must target the astrocyte-tumor axis. This section outlines emerging precision strategies, from pharmacological reprogramming and immunotherapy to advanced gene editing and nanomedicine.

### Pharmacological reprogramming: "Normalizing" the TME

Rather than indiscriminately ablating all astrocytes—a strategy that would disrupt CNS homeostasis—a more sophisticated approach is to pharmacologically “re-educate” reactive astrocytes, reverting them from a tumor-promoting to a quiescent or tumor-suppressive state.

#### Anti-inflammatory and STAT3 inhibitors

Given the centrality of the JAK/STAT3 pathway, STAT3 inhibitors are prime therapeutic candidates. Small-molecule inhibitors (e.g., WP1066) and natural compounds (e.g., silibinin) can block STAT3 phosphorylation in reactive astrocytes, reducing MMP secretion and downregulating PD-L1 and TRAIL [34, 168]. Beyond direct STAT3 inhibition, clinical repurposing of drugs like Celecoxib, a selective COX-2 inhibitor, can dampen the NF-κB-driven inflammatory phenotype and has shown synergistic effects with temozolomide [51, 169]. Other potential strategies include the targeted inhibition of NF-κB subunits (IKKβ or RelA) [170]. inhibition of SIRT1 to switch reactive astrocytes to an anti-inflammatory phenotype [171], and activation of the pregnane X receptor (PXR) using rifampicin to tighten the BBB [172, 173].

#### Gap junction blockade: severing the supply lines

Disrupting the physical coupling between astrocytes and tumor cells is a promising strategy to starve tumors of metabolic and survival support. Tonabersat, originally developed for migraine, acts as a brain-penetrant drug that specifically blocks Cx43 gap junctions. By severing the metabolic connection through which astrocytes provide calcium buffering and mitochondrial transfer, Tonabersat sensitizes GBM cells to temozolomide and dismantles the resistance network [174]. Unlike general blockers, peptide inhibitors such as αCT1 specifically disrupt the interaction between Cx43 and the cytoskeletal protein ZO-1. This targeted interference inhibits glioma cell motility along astrocytic processes and reduces the formation of invasive “leading edges” [175].

Emerging evidence indicates that Cx43 promotes glioblastoma progression through both channel-dependent exchange and channel-independent signaling via its C-terminal tail. Notably, Cx43 can scaffold PI3K signaling to confer chemoresistance, implying that blocking conductance alone may be insufficient [36]. Consistent with this, glioma-astrocyte Cx43 coupling has been linked to temozolomide resistance through a defined miRNA-transcriptional DNA repair axis (miR-205-5p/E2F1/ERCC1), and genetic or pharmacological inhibition of Cx43 restores chemosensitivity [176].

From a translational perspective, CNS-penetrant repurposed agents and dedicated gap junction modulators are both being explored. Chlorpromazine has been reported to reverse temozolomide resistance by inhibiting Cx43 and concomitantly suppressing DNA repair pathways [177], and tonabersat can enhance temozolomide-mediated cytotoxicity by disrupting Cx43-mediated intercellular connectivity in glioblastoma models [178]. More recently, hemichannel-targeting biologics (for example abEC1.1) have shown preclinical efficacy in suppressing glioblastoma progression while reducing tumor-associated hyperexcitability, offering an additional therapeutic entry point beyond classical gap junction blockade [179]. Future efforts should prioritize delivery strategies that concentrate inhibitors at the tumor-astrocyte contact zone to maximize efficacy while minimizing disruption of physiological astrocytic networks.

### Immunotherapies targeting the glial shield

**Fig. 5:**
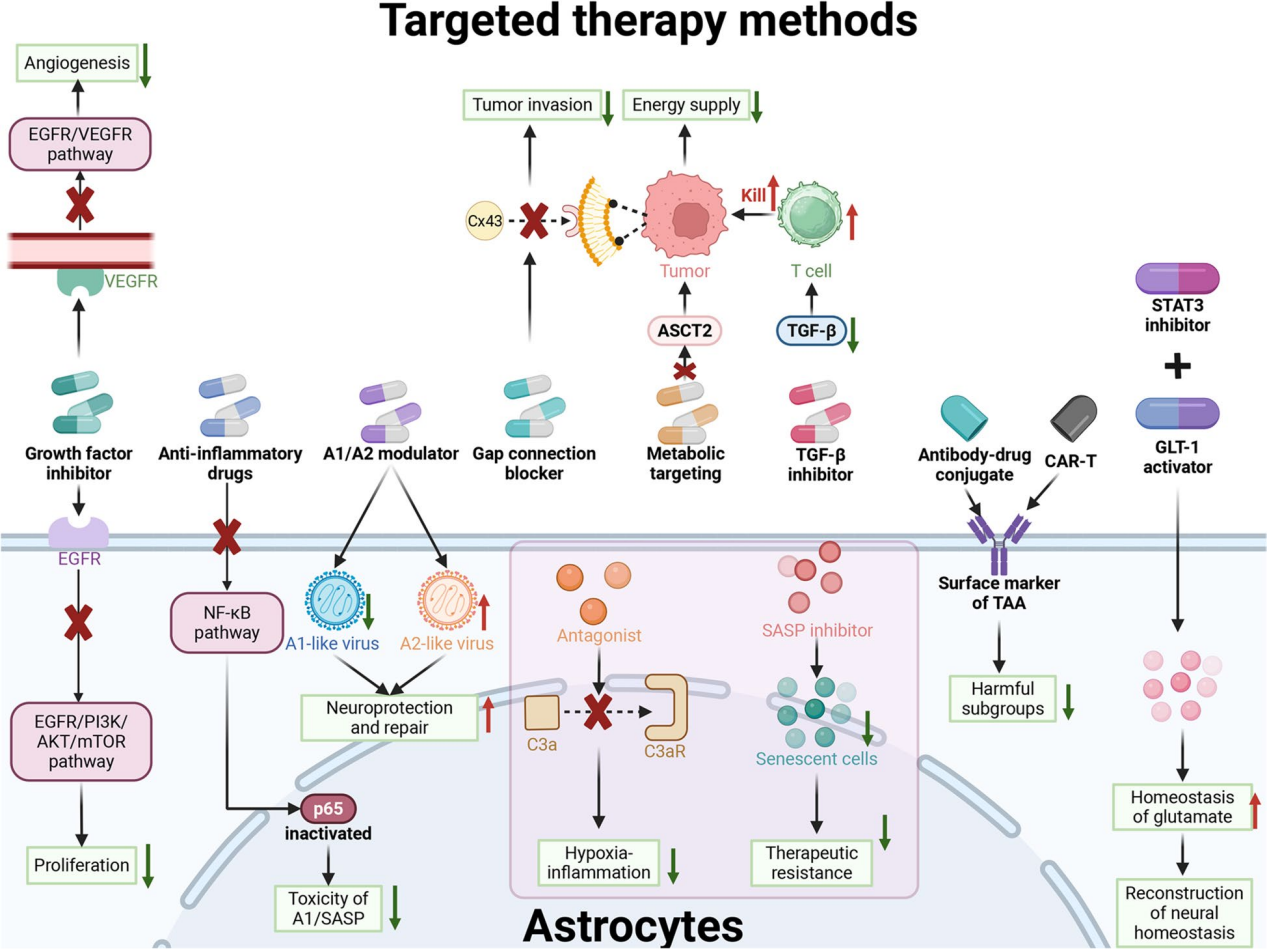
Current and emerging therapeutic strategies for targeting astrocytes: mechanisms and challenges. Therapeutic strategies designed to counteract the protumorigenic functions of astrocytes are broadly categorized into pathway inhibition and functional reprogramming. Current broad-spectrum approaches often involve inhibiting major growth factor pathways, such as EGFR or VEGF, or employing general anti-inflammatory agents. Emerging precision strategies aim for greater specificity by blocking direct communication with tumor cells via connexin-43 gap junction inhibitors, disrupting metabolic support through ASCT2 transporter inhibition, or neutralizing specific inflammatory pathways using C3a receptor antagonists. Looking forward, advanced approaches focus on reprogramming astrocytes away from a harmful state—such as modulating A1/A2 phenotypes or inhibiting the senescence-associated secretory phenotype (SASP)—and restoring homeostatic functions like GLT-1 activity. The overarching goal is to employ highly specific modalities, including antibody–drug conjugates (ADCs) or chimeric antigen receptor (CAR) T cells, to selectively ablate pathogenic astrocyte subsets and inhibit tumor progression while preserving essential central nervous system functions. This figure was created with BioRender.com. Abbreviations: EGFR, epidermal growth factor receptor; VEGF, vascular endothelial growth factor; VEGFR, vascular endothelial growth factor receptor; PI3K, phosphoinositide 3-kinase; AKT, protein kinase B; mTOR, mechanistic target of rapamycin; NF-κB, nuclear factor-kappa B; Cx43, connexin-43; ASCT2, alanine-serine-cysteine transporter 2; TGF-β, transforming growth factor-beta; C3aR, C3a receptor; SASP, senescence-associated secretory phenotype; STAT3, signal transducer and activator of transcription 3; GLT-1, glutamate transporter-1; ADC, antibody–drug conjugate; CAR-T, chimeric antigen receptor T cell; TAA, tumor-associated astrocyte; CNS, central nervous system

Standard immune checkpoint inhibitors (e.g., anti-PD-1) often fail in GBM because the “glial shield” prevents T-cell access. Next-generation immunotherapies are designed to penetrate or dismantle this barrier by targeting surface markers enriched on TAAs (Fig. 5).

#### Chimeric Antigen Receptor (CAR) T-cell therapy

While first-generation CAR-T cells targeting single antigens showed limited durability due to antigen escape, novel designs address TME heterogeneity by simultaneously targeting the supportive stroma [180]. Recent phase I trials (e.g., NCT05168423) using Tandem CAR (TanCAR) T cells co-targeting IL-13Rα2 and EGFRvIII have achieved breakthroughs. IL-13Rα2 is upregulated on a subset of reactive astrocytes. TanCAR T cells thus act as dual-purpose effectors, eliminating tumor cells while clearing the immunosuppressive astrocytic niche. Intrathecal delivery led to rapid tumor regression in 62% of patients, with some achieving durable complete responses, suggesting stromal targeting prevents antigen escape [175]. Another strategy is the use of Chlorotoxin-CAR T (CLTX-CAR) cells, which utilize a scorpion venom-derived peptide to bind membrane-bound MMP-2. As MMP-2 is highly upregulated on invasion-leading astrocytes but absent in the normal brain, this enables precise targeting of the invasive front [181, 182]. To overcome the BBB, delivery strategies have shifted towards intracranial or intrathecal administration, ensuring high effector concentrations directly at the astrocyte-tumor interface. [181, 182].

#### Antibody–Drug Conjugates (ADCs)

ADCs enable the targeted delivery of cytotoxic payloads to specific cell types by turning astrocytic surface markers against them. One approach involves targeting Glypican-1 (GPC1), a cell-surface proteoglycan highly enriched on TAAs and GBM cells but largely absent in the normal adult brain. Anti-GPC1 ADCs are conjugated with microtubule disruptors (e.g., MMAE). Upon binding and internalization, the cytotoxic payload is released to induce apoptosis. In orthotopic models, this strategy of stromal depletion potently inhibited tumor growth and extended survival by eliminating the protective astrocytic scaffold [183]. Additionally, EVA1-targeting ADCs have shown promise in eliminating tumor-initiating cells, representing another selective approach [184].

### Gene therapy and RNA interference: precision silence

RNA interference (RNAi) and gene editing offer high specificity to silence “undruggable” targets or correct dysregulated pathways within astrocytes. The central challenge remains efficient and specific delivery across the BBB.

#### Lipid Nanoparticles (LNPs) and receptor-mediated delivery

Advanced nanocarriers protect nucleic acids and facilitate BBB traversal [185]. Nanoparticles functionalized with the Angiopep-2 peptide utilize LRP1, which is highly expressed on BBB endothelial cells and reactive astrocytes. These nanoparticles cross the BBB via receptor-mediated transcytosis and are subsequently internalized by astrocytes [186]. They have successfully delivered siRNA against STAT3 or astrocyte elevated gene-1 (AEG-1). Silencing AEG-1 inhibits the NF-κB pathway and reduces the secretion of CXCL1 and IL-6, thereby curbing tumor invasion and angiogenesis.

Beyond proof-of-concept cargo delivery, siRNA-LNP platforms are particularly relevant for astrocyte targeting for two practical reasons. First, they enable transient and titratable suppression of upstream regulators that define tumor-associated astrocyte programs, which aligns with normalization strategies aiming to reprogram astrocytes rather than ablate them. Second, their implementation can be adapted to central nervous system constraints by matching the delivery route to the clinical objective: local administration (intratumoral injection or convection-enhanced delivery) to maximize parenchymal exposure at the astrocyte–tumor interface, CSF-facing administration (intraventricular or intrathecal) to interrogate perivascular and ventricular compartments, and systemic delivery only when paired with receptor-mediated targeting ligands. In this context, a state-aware implementation framework should predefine(i)the astrocyte program to be shifted (for example,barrier-associated,inflammatory,immunosuppressive,or metabolic stress programs),(ii)the regulatory node to silence (such as STAT3 or other program-defining factors),and(iii)the biomarker panel used to verify on-target astrocyte reprogramming in vivo.

#### MicroRNA replacement therapy

Nanomedicine can be utilized to restore tumor-suppressive microRNAs downregulated in reactive astrocytes. Doxorubicin-loaded engineered nanocarriers (Nano-DOX) represent one strategy that not only kills tumor cells but also blocks the IL-6/STAT3 feedback loop between GBM cells and astrocytes. This disruption disconnects the symbiotic relationship and reverts astrocytes to a less supportive state [187]. Alternatively, the systemic delivery of miR-124 mimics encapsulated in nanoparticles can inhibit STAT3 signaling. This approach helps revert astrocytes to quiescence and sensitizes the TME to chemotherapy [188].

#### CRISPR/Cas9 genome editing

Although in early preclinical stages, vivo gene editing represents a powerful tool for TME remodeling. Using adeno-associated virus (AAV) vectors with astrocyte-specific promoters (e.g., gfaABC1D), CRISPR/Cas9 machinery can be delivered directly to astrocytes. Designing sgRNAs to target the Cd274 (PD-L1) locus enables the permanent excision of PD-L1 from the astrocytic genome. In murine glioma models, this genetic intervention significantly enhanced CAR-T cell therapy efficacy, leading to long-term survival [189, 190].

### Stem cell-based therapies: the "Trojan Horse" strategy

Neural stem cells and mesenchymal stem cells (MSCs) possess intrinsic tumor-homing ability, migrating along chemokine gradients (e.g., CXCL12/CXCR4 axis) to locate glioma deposits.

#### Enzyme/prodrug systems (Suicide Gene Therapy)

This advanced application utilizes stem cells as vehicles to deliver suicide enzymes. Neural stem cells engineered to express bacterial cytosine deaminase (CD) migrate to tumor cores and invasive margins. Upon systemic administration of the prodrug 5-fluorocytosine (5-FC), which can pass the BBB, the NSC-derived CD converts it into the potent chemotherapeutic 5-fluorouracil (5-FU) within the TME. The small-molecule 5-FU diffuses to kill neighboring tumor cells and reactive astrocytes locally with minimal systemic toxicity. Clinical trials (e.g., NCT01172964) have validated the safety and feasibility of this approach [191].

#### Oncolytic virus delivery

Stem cells can also shield oncolytic viruses (OVs) during transport to overcome rapid immune clearance. Neural stem cells loaded with CRAd-S-pk7, a conditionally replicating adenovirus driven by the survivin promoter, home to tumors. The subsequent virus release at the tumor site infects and lyses survivin-positive tumor cells and reactive astrocytes. This process not only reduces the tumor burden but also releases tumor antigens, triggering a secondary anti-tumor immune response. A phase I trial (NCT03072134) is currently evaluating this strategy in high-grade glioma [192].

#### Astrocyte replacement/engineering

A more futuristic concept involves transplanting healthy, engineered glial progenitor cells (GPCs). These GPCs would differentiate into “super-astrocytes” genetically modified to resist tumor-derived “education” signals (e.g., lacking IL-11R or TGF-β receptors). By replacing corrupted host stroma with resistant cells, this approach aims to restore glutamate buffering and BBB integrity, effectively “normalizing” the TME from the ground up [193].

### Conclusion: towards a Holobiont treatment

The complexity of the astrocyte-glioma interaction—spanning metabolic coupling, immune evasion, and physical integration—underscores that brain tumors cannot be treated as isolated masses of malignant cells. They are complex, adaptive “organs of disease.” The future of neuro-oncology lies in combinatorial therapies: using surgery and radiation to debulk the tumor mass, while simultaneously deploying astrocyte-targeted agents (e.g., STAT3 inhibitors, Cx43 blockers) to dismantle the supportive scaffold. By turning the “soil” (astrocytes) against the “seed” (tumor), we may finally overcome the therapeutic resistance that defines this devastating disease (Table 3).

**Table 3:** Therapeutic strategies targeting astrocytes: mechanisms, benefits, and challenges

Strategy category (example agents)	Mechanism/Target	Potential benefit	Key challenge(s)	Current stage	ClinicalTrials.govID(NCT)	Reference
Gap junction blocker	Cx43 (Tonabersat, αCT1)	Severing metabolic supply (Ca^2+^ buffering, mitochondria); Reducing invasion	Specificity to tumor-astrocyte coupling	Preclinical (Tonabersat repurposed)	Not applicable (preclinical)	[89, 175]
Anti-inflammatory/STAT3	STAT3 inhibitors (WP1066, Silibinin), Celecoxib	Downregulates PD-L1/TRAIL (Restores immunity), Reduces edema	Complexity of inflammatory signaling	Preclinical/Clinical (Celecoxib)	NCT00068770	[34, 42, 43, 168, 169]
SASP inhibitor	Targeting senescent cells	Reduces pro-tumorigenic secretome (IL-6, HGF) post-radiation	Specific targeting of senescent TAAs	Preclinical	Not applicable (preclinical)	[194]
Specific TAA Subtype Ablation	ADC (Anti-GPC1), CAR-Ts (EGFRvIII, IL13Rα2, CLTX as examples)	Stromal depletion; Targeting invasive edge (MMP-2) or immunosuppressive niche	Identification of exclusive surface targets	Preclinical/Phase I Trials	EGFRvIII: NCT02664363; NCT02844062; NCT03726515; IL13Rα2: NCT06815029; NCT06355908; CLTX: NCT04214392	[181, 182, 185]
Gene Therapy/RNAi	LNP-delivered siRNA (Ang-LNP against STAT3/AEG-1), CRISPR (PD-L1 knockout)	Dismantling immune evasion hubs or inflammatory drivers	BBB delivery efficiency	Preclinical	Not applicable (preclinical)	[186, 187, 190]
Stem Cell-Based	NSC/MSC (Enzyme/Prodrug: CD/5-FC), Oncolytic Virus delivery	"Trojan Horse" delivery of chemotherapy or viruses to tumor core/margin	Clinical Trials	Clinical Trials	CD/5-FC: NCT01172964; NCT04657315; NCT07143812 Oncolyticvirusdelivery(NSC-CRAd-S-pk7): NCT03072134; NCT05139056; NCT06169280	[181, 192]
Functional Reprogramming	PXR activation (Rifampicin), miR-124 mimics	Restoration of BBB integrity, Reversion to quiescent phenotype	Monitoring functional recovery	Conceptual/Preclinical	Not applicable (preclinical)	[173, 189]
Adjuvant targets	Gut-Brain Axis (Dietary Tryptophan/AhR)	Dampening neuroinflammation via systemic metabolic modulation	Patient compliance, Microbiome variability	Conceptual/Emerging	Not applicable (preclinical)	[38, 39]

## Conclusions and future perspectives

Reactive astrogliosis has long been viewed through a reductionist lens, often dismissed as a uniform “scarring” response secondary to tumor growth. However, the comprehensive evidence synthesized here compels a fundamental paradigm shift: astrocytes are not passive bystanders but active, dynamic architects of the brain tumor ecosystem.

Throughout this review, we have delineated how the heterogeneity–plasticity–duality triad defines the astrocytic contribution to malignancy. First, regarding heterogeneity, we have moved beyond the simplistic A1/A2 binary to recognize a multidimensional spectrum of states—from the MES-hypoxia population driving angiogenesis in the core to the MMP-high phenotypes guiding invasion at the margin. Second, astrocytes exhibit remarkable plasticity, epigenetically rewiring themselves via EZH2 and HIF-1α to meet tumor demands. They function as “energy reservoirs,” transferring lactate and mitochondria to rescue metabolically impaired glioma cells. Finally, and most critically, we highlight the duality of astrocytes. While possessing an intrinsic capacity to contain tumors via scarring, this defense is systematically hijacked, as the tumor “educates” astrocytes to turn their shields into weapons for immune evasion and therapeutic buffering.

While we have mapped the broad contours of the astrocyte-tumor interaction, translating this knowledge into clinical benefit requires navigating key unknowns. We propose three interconnected pillars for future investigation.

Future research must leverage spatial transcriptomics (e.g., MERFISH, Visium) combined with longitudinal intravital imaging to create 4D maps of astrocyte evolution. Crucially, this mapping should focus on capturing the "inflection point" where astrocytes transition from a defensive, scar-forming phenotype (characterized by high GFAP, STAT3 phosphorylation, and tight junction integrity) to a pro-tumorigenic, mesenchymal state (marked by CD44, TNC, and SERPINE1 expression). Emerging evidence suggests that this switch is not binary but graded, likely driven by a signaling threshold where cumulative tumor-derived TGF-β and hypoxia-induced HIF-1α signaling overwhelms the homeostatic STAT3-mediated containment program, triggering NF-κB-dependent remodeling. Understanding this spatiotemporal trajectory is essential for determining the optimal window for astrocyte-targeted interventions.

A practical roadmap for a glial liquid biopsy requires a strict compartmentalization of analytes. First, tumor-derived circulating tumor DNA (ctDNA) should be utilized to track the genomic evolution of the malignant "seed," offering insights into mutational burden and clonal heterogeneity [195, 196]. Second, and distinct from tumor markers, the "soil" status must be monitored via astrocyte-derived extracellular vesicles (ADEVs). Unlike bulk CSF analysis [197], immunocapture workflows enriching for GLAST + or GLT-1 + vesicles enable the specific isolation of astrocytic cargo [198]. Profiling these ADEVs for reactive miRNAs (e.g., miR-19b) or metabolic enzymes can provide a real-time readout of the stromal switch from containment to support, independent of tumor volume.

The limitations of broad-spectrum ablation underscore that eliminating astrocytes is detrimental to brain health. The future therefore lies in stromal normalization, namely reprogramming tumor-associated astrocytes toward more homeostatic outputs while preserving essential barrier and metabolic functions. Within this concept, gene therapy and RNA interference are especially attractive because siRNA-LNP platforms can provide transient and titratable suppression of upstream state-defining regulators, enabling reprogramming rather than irreversible depletion. A practical implementation requires matching delivery routes to the objective, including local administration (intratumoral injection or convection-enhanced delivery) to maximize exposure at the astrocyte–tumor interface, CSF-facing administration (intraventricular or intrathecal) to interrogate perivascular and ventricular compartments, and systemic delivery only when coupled to receptor-mediated targeting ligands. Critically, normalization strategies should be evaluated against predefined astrocyte program endpoints, specifying which program is intended to shift and which biomarker panel will verify on-target state changes in vivo. This approach can be complemented by systemic modulators, such as gut–brain axis interventions that engage astrocytic AhR signaling, as a low-toxicity adjuvant to dampen protumorigenic neuroinflammation.

Brain tumors can no longer be conceptualized as isolated masses of malignant cells; they are complex organs of disease, critically supported by a corrupted glial scaffold. By shifting the therapeutic crosshairs to include the astrocyte—targeting its metabolic fuel lines, dismantling its immune shields, and severing its physical connections—we open a new dimension of therapeutic opportunity. The ultimate goal is no longer merely to kill the seed, but to reclaim and rehabilitate the soil.

## Data Availability

Not applicable.

## Abbreviations

CNS: Central nervous system
BBB: Blood-brain barrier
BTB: Blood-tumor barrier
GBM: Glioblastoma
BrM: Brain metastases
TME: Tumor microenvironment
TAA: Tumor-associated astrocyte
GM: Gray matter
WM: White matter
WHO: World Health Organization
AQP4: Aquaporin-4
NVU: Neurovascular unit
EAAT1/2: Excitatory amino acid transporters 1 and 2
TAMs: Tumor-associated macrophages
MDSCs: Myeloid-derived suppressor cells
ECM: Extracellular matrix
MMPs: Matrix metalloproteinases
CSPGs: Chondroitin sulfate proteoglycans
2-HG: D-2-hydroxyglutarate
IDH: Isocitrate dehydrogenase
TLRs: Toll-like receptors
IL-6: Interleukin-6
STAT3: Signal transducer and activator of transcription 3
JAK/STAT3: Janus kinase/signal transducer and activator of transcription 3
NF-κB: Nuclear factor-kappa B
PD-L1: Programmed death-ligand 1
TRAIL: TNF-related apoptosis-inducing ligand
IL-1R: Interleukin-1 receptor
TNFR: Tumor necrosis factor receptor
MyD88: Myeloid differentiation primary response 88
MAPK: Mitogen-activated protein kinase
ERK: Extracellular signal-regulated kinase
NFAT: Nuclear factor of activated T cells
GFAP: Glial fibrillary acidic protein
VEGF: Vascular endothelial growth factor
PVAs: Perivascular astrocytes
TNC: Tenascin-C
ASCT2: Alanine-serine-cysteine transporter 2
MCT1/4: Monocarboxylate transporters 1 and 4
PAs: Plasminogen activators
DR5: Death receptor 5
cGAS-STING: Cyclic GMP-AMP synthase-stimulator of interferon genes
SASP: Senescence-associated secretory phenotype
EZH2: Enhancer of zeste homolog 2
TET: Ten-eleven translocation
AhR: Aryl hydrocarbon receptor
SHH: Sonic hedgehog
AChE: Acetylcholinesterase
Cx43: Connexin-43
TNTs: Tunneling nanotubes
EVs: Extracellular vesicles
ADEVs: Astrocyte-derived extracellular vesicles
ORM2: Orosomucoid-2
RGD: Arginine-glycine-aspartate
SVZ: Subventricular zone
scRNA-seq: Single-cell RNA sequencing
siRNA: Small interfering RNA
CAR-T: Chimeric antigen receptor T cells
ADCs: Antibody-drug conjugates
LNPs: Lipid nanoparticles
RNAi: RNA interference
TMZ: Temozolomide
CSF: Cerebrospinal fluid
